# Knowing our value and our values: toward an ethical practice of library assessment

**DOI:** 10.29173/jchla29941

**Published:** 2026-08-03

**Authors:** Jeff Mason

**Affiliations:** Innovation and Entrepreneurship, Health Sciences Library, McMaster University, Hamilton, ON, Canada

Young SWH. **Knowing our value and our values: toward an ethical practice of library assessment**. Sacramento, CA: Library Juice Press; 2024. Softcover: 256 p. ISBN: 978-1-63400-143-4. Price: USD$45.00. Available from: https://litwinbooks.com/books/knowing-our-value-and-our-values/.

## Overview and summary

Demonstrating value and impact has been a topic of interest for health librarians, particularly those supporting clinical care, since at least 1992 and the publication of Marshall’s Rochester study [1]. Since then, assessing value and impact has been revisited by Canadian health librarians across the country [2–4] and has also been articulated in CHLA/ABSC’s standards and the association’s Library Value Planner tool [5–7]. In addition to demonstrating our value as health librarians, health libraries are also guided by professional sets of values articulated implicitly through vision and mission statements (e.g., CHLA/ABSC [8]), explicitly as values statements (e.g., ALA [9]) , or as a hybrid of both (e.g., MLA [10]). We are also influenced by the values of our parent organizations and politics of the day. Navigating how we demonstrate value in the face of ethical dilemmas that arise when assessment conflicts with our values is the subject of *Knowing our value and our values: towards and ethical practice of library assessment*.

*Knowing our value and our values* presents original research by Scott Young, a user-experience and assessment librarian at Montana State University with professional and research interests in participatory design, professional self-reflection, and professional ethics [11]. With the goal of answering the question, “How can library assessment be practiced ethically?”, Young explores the challenges that arise when librarians make decisions in diverse, varied, and contextually dependent settings where individual, professional, and institutional values conflict (e.g., patron privacy versus collection usage) and presents new tools to help readers navigate decision-making in the ethically challenging landscape of library assessment.

The book begins with an overview chapter introducing the problem, methods, and outputs of Young’s research. Chapters two through four establish the values and practices of library assessment and introduce key concept used in the research (e.g., practical ethics, constructivist grounded theory). Research results (survey and interview findings with assessment librarians) are presented in chapters five and six and research outputs—the *Values classification for library assessment* and the *Values-sensitive library assessment toolkit*—are discussed in Chapter six. The values classification and toolkit represent the book’s main contributions to the topic of ethical library assessment and the toolkit is available to readers on Open Science Framework [12].

*Knowing our value and our values* concludes with a chapter summarizing Young’s research and looking to the future of values-based library assessment. This final chapter is a good entry point to Young’s research, the values classification, and the toolkit. Readers without time to read the whole book, or with a passing interest in library assessment, could consider reading the summary after the overview and leave with a strong understanding of Young’s research and its implications for their practice.

## Content and idea

While, *Knowing our value and our values* is written with North American academic assessment librarians in mind, the book’s content and tools are applicable to librarians, library workers, and library administers in other settings, such as health libraries. Readers with an interest in the topics discussed in the book from a Canadian academic library perspective may find Bond’s review in the *Canadian journal of academic librarianship* helpful for understanding the book’s application in that context [13].

Young’s ideas are presented in sufficient detail for readers familiar with, or new to, the main topics underpinning his research into the values of assessment librarians and the practice of ethical and values-based library assessment (i.e., history of library values, constructivist grounded theory, and practical ethics). The writing is clear and accessible, and the book is largely free of errors (the one or two typos encountered could be easily revised in an updated edition). Footnotes and references are available throughout and connected to the body of the text.

Each chapter of *Knowing our value and our values* builds thoughtfully upon the last. This structure is helpful for readers who may be new to library assessment, library values, or practical ethics. Young is also mindful to provide examples and scenarios for what could easily become entirely theoretical or theory heavy content. For example, chapter three “Sites of tension for ethical decision-making” begins with an overview of two major types of ethical dilemmas (competing external values systems and competing internal values systems) before literature-informed discussions of specific sites of tension (e.g., values studies and impact assessment, cataloguing and classification). The examples and scenarios are diverse enough that most readers should be able to find at least one that resonates with their own experience and context.

**Fig. 1:**
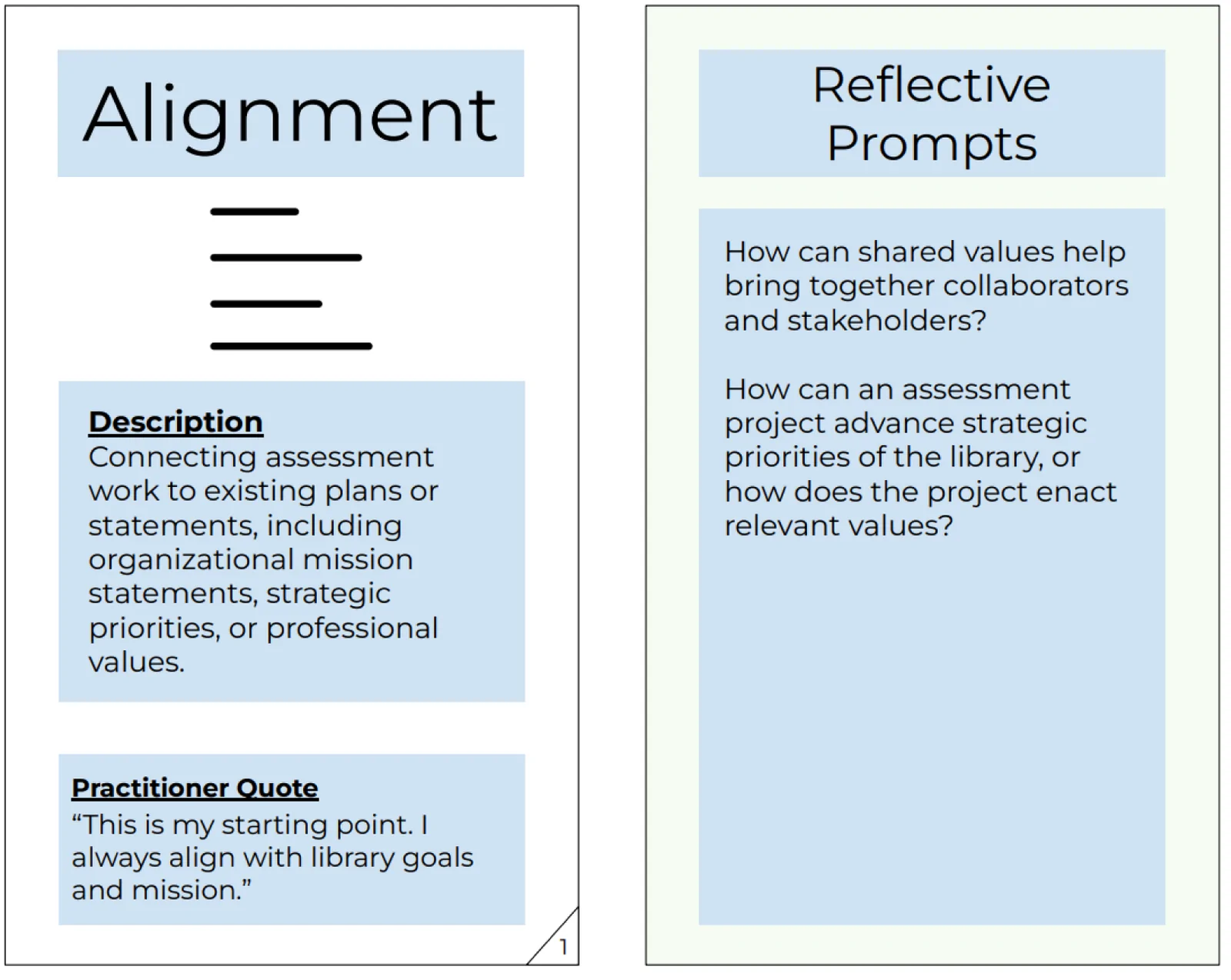
An example of a card from the *Values-sensitive library assessment toolkit*. One side of the card includes the name of the value (alignment), a description, and a quote from an assessment librarian. The other side of the card includes reflective prompts that can be used in groups or individually.

**Fig. 2:**
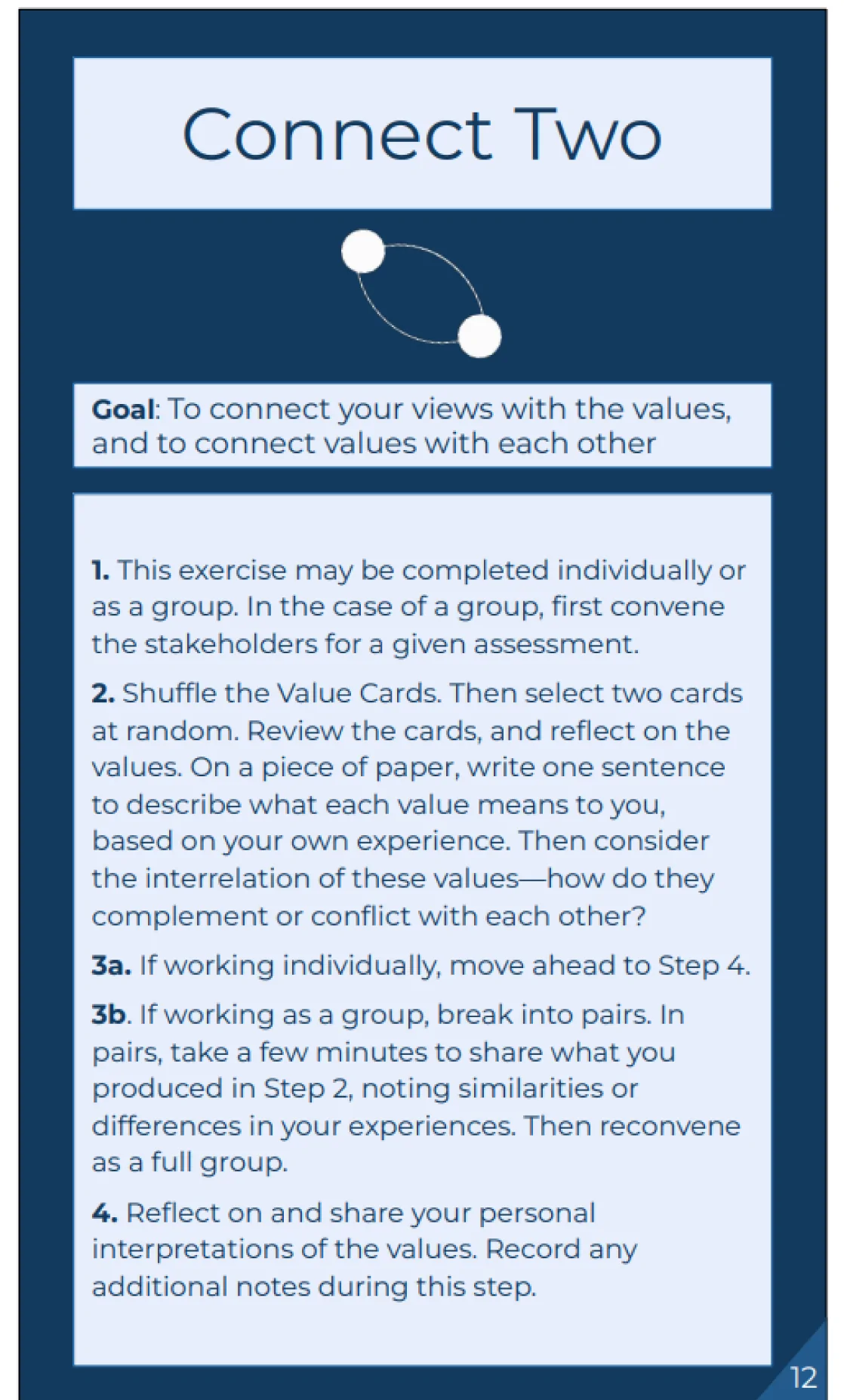
An example of an exercise card from the *Values-sensitive library assessment toolkit*. Each exercise card includes the name of the exercise, its goal, and instructions for completing the exercise in groups or individually.

*Knowing our value and our values* makes two main contributions to library assessment, the *Values classification for library assessment* (a set of 10 values informed by the literature and a survey of assessment librarians) and the *Values-sensitive library assessment toolkit* (available online through Open Science Framework at: doi.org/10.17605/OSF.IO/ZS5C8). Designed as an expandable set of playing cards (Figures 1 and 2), the toolkit offers readers a practical way to apply the *Values classification* to their own library assessment practices. The toolkit’s content extends logically from the research and ideas discussed in the book and Young’s rationale for its design [14] (e.g., allowing for consideration of multiple, conflicting values, including blank cards to adapt the toolkit to local contexts, developing exercises informed by participatory design) aligns well with Young’s research.

Young’s point of view as a researcher is directly addressed in the book and reflections on positionality are included in the opening chapter [15] and closing chapter [16]. Clear statements of positionality and self-reflection help the reader understand what part of the book’s content are most applicable to their work and how the values classification and toolkit may need to be adapted to work in their own setting.

## Applicability to Canadian health libraries

Throughout *Knowing our value and our values*, Young explores tensions that exist between value systems and the need to demonstrate our value as library workers and libraries. For Canadian health librarians, the conflict between our values and market forces [17] will ring particularly true. Arguing that libraries and the public sector organizations that fund them are increasingly influenced by capitalist values (e.g., competition, risk, value for money, information as a commodity), Young states librarians may act against our professional and personal values, creating or employing measures of success (e.g., student retention, collection of personal data) that align more with market values than our own values. Most health librarians, especially those working in hospital, government, nonprofit, or private sector settings, will have experienced and navigated these pressures in their work—for example, tracking resource use may conflict with patron privacy, but is necessary to demonstrate return on investment for the organization. By articulating how the world we live and work in creates these tensions, creating a practical and ethical path to resolving them in a way aligned with our values, *Knowing our value and our values* is a helpful resource for health librarians trying to demonstrate value to funders.

## Concluding remarks

In the face of continued existential threats to health libraries and health library workers [18], demonstrating our value is more important than ever. *Knowing our value and our values*, offers readers a well-researched, accessible, and practical approach to assessing the work we do to support health education, health policy, health research, and patient care in an ethical, values-centred way.
